# P-2040. Assessment of the Relationship of Medical Comorbidity to Graduation from an HIV Care Coordination Program

**DOI:** 10.1093/ofid/ofaf695.2204

**Published:** 2026-01-11

**Authors:** Nicholas F Yared, Smitha Gudipati, Brianna Hohmann, Victoria Warzocha, Shannon Payne, Indira Brar

**Affiliations:** Henry Ford Health System, Detroit, MI; Henry Ford Health System, Detroit, MI; Henry Ford Health, Detroit, Michigan; Henry Ford Health, Detroit, Michigan; Henry Ford Health, Detroit, Michigan; Henry Ford Hospital, Detroit, Michigan

## Abstract

**Background:**

People with HIV (PWH) face a great burden of developing chronic comorbidities impacting care. Multiple studies have shown a negative association between increased comorbidity burden and antiretroviral therapy (ART) adherence. Henry Ford Care Coordination Program (CCP) provides intensive case management services to PWH facing barriers with achievement of viral load suppression (VS). Durable VS for at 1 year allows for graduation from the program. We aimed to assess if having more comorbidities impacted care coordination program completion.

**Methods:**

A retrospective study reviewing co-morbidities of patients enrolled in the CCP between 2019 and 2024. Data abstraction through chart review was used to calculate Charlson Comorbidity Index (CCI) for each patient. CCI values were categorized as low (0-2), moderate (3-4), or high (≥5). Advanced HIV status (CD4+ count < 200 cells/μL) was recorded at graduation or early discharge from the program. Graduation was defined as achieving HIV-1 VL < 200 copies/mL at 1 year. If viral suppression was not achieved at 1 year or PWH did not engage in the program, they were discharged. Chi square tests were used to analyze the relationship between CCI and graduation and CCI and advanced HIV status.

**Results:**

Of 141 PWH enrolled in the CCP, 41 (29%) graduated, 95 (67%) were discharged, and 5 (4%) died over the analysis period. The 136 living patients had average age of 44 years (SD 12.8) and were 79% male, 69% black race, and 24% advanced HIV. CCI was 0-2 for 85 patients (63%), 3-4 for 13 patients (10%), and ≥5 for 38 patients (28%). Chi square analysis did not show an association between CCI and CCP graduation (Χ^2^ 4.1, df 2, p = 0.13) but did show an association between advanced HIV status and CCP graduation (Χ^2^ 4.7, df 1, p = 0.03). Only 5 PWH with advanced status (4%) graduated from the program.

**Conclusion:**

Advanced HIV status is a component of CCI, and this patient subgroup was less likely to achieve VS. Overall comorbidities, however, as reflected by CCI were not identified as a potential barrier for patients to achieve VS in our study. Identifying interventions that tailor care coordination for patients with advanced HIV would likely positively impact care.

**Disclosures:**

Indira Brar, MD, Gilead: Advisor/Consultant|Gilead: Grant/Research Support|Gilead: Honoraria|ViiV Healthcare: Advisor/Consultant|ViiV Healthcare: Grant/Research Support|ViiV Healthcare: Honoraria

